# The Influence of Process Conditions and Reinforcement Characteristics on the Densification and Mechanical Properties of Powder Metallurgy SiC_p_/Al Composites

**DOI:** 10.3390/ma18215060

**Published:** 2025-11-06

**Authors:** Liuxu Cao, Qingsong Dai, Qiwen Liang, Xiaoyong Zhang

**Affiliations:** 1 State Key Laboratory of Powder Metallurgy, Central South University, Changsha 410083, China; 2 Light Alloy Research Institute, Central South University, Changsha 410083, China; 3 School of Materials Science and Engineering, Kunming University of Science and Technology, Kunming 650093, China

**Keywords:** aluminum matrix composites, powder metallurgy, densification, microstructure

## Abstract

Compared to pure aluminum powder, aluminum alloy powders exhibit higher strength and hardness, which leads to greater difficulty in plastic deformation during cold compaction, consequently impairing green compact formation and subsequent densification. This study introduces pure aluminum powder into the raw material system based on 2024 aluminum alloy powder and SiC powder, effectively improving the powder compaction characteristics. A systematic investigation was conducted to examine the effects of sintering temperature (460–640 °C) and holding time (5–120 min) during pressureless sintering on the sintering shrinkage, relative density, mechanical properties, and microstructure of SiC_p_/Al composites reinforced with 35 wt.% of 31.9 μm SiC particles. The results indicate that sintering at 600 °C for 30 min constitutes the optimal process condition, achieving effective interparticle bonding while preventing coarsening of both precipitates and pores. Subsequent hot pressing effectively enhanced the relative density and mechanical properties of the sintered preforms, achieving a maximum tensile strength of 343 MPa, which represents an improvement of over 70% compared to the sintered-only state. For the hot-pressed state, elevated levels of SiC particle content and size compromised its mechanical performance. This work demonstrates a highly operable and industrially viable processing route for manufacturing aluminum matrix composites using alloy powders.

## 1. Introduction

SiC_p_/Al composites possess advantages such as lightweight with high strength, high thermal conductivity, excellent wear resistance, and low thermal expansion, making them widely applicable in aerospace [1,2], transportation equipment [3,4], electronics [5], and other fields. For example, the 12 vol.% SiC_p_/2A14 aluminum matrix composite can achieve a tensile strength of over 450 MPa after heat treatment and has been successfully applied in the structural framework of the space station’s solar battery array [1]. 2XXX series aluminum alloys are preferred matrix materials for SiC_p_/Al composites due to their superior mechanical properties and temperature resistance and AA 2024 is a notable example [6,7,8]. With the addition of SiC, the SiC_p_/2024Al composites demonstrated enhanced mechanical properties and superior electrochemical corrosion resistance [7]. Among various manufacturing techniques for aluminum matrix composites, powder metallurgy is the typical preparation method for SiC_p_/Al composites due to its advantageous material design flexibility and excellent comprehensive properties [9].

The powder metallurgy process encompasses a broad spectrum of techniques, and the selection of a specific processing route depends not only on material performance requirements but is also closely related to raw material characteristics. Aluminum alloy powder offers the advantage of inherent homogeneous elemental distribution from the production stage, which simplifies the mixing process. However, a critical challenge in powder processing arises from the relatively poor compaction performance of aluminum alloy powders compared to pure aluminum, primarily due to their higher strength and work-hardening tendency. Therefore, when aluminum alloy powder is selected as the raw material, advanced forming methods such as hot isostatic pressing (HIP) [10], hot-press sintering [11], hot extrusion [12,13], or thixoforming [14] are typically employed. Song [15] et al. fabricated SiC_p_/2024Al composites reinforced with 10 wt.% nano-sized SiC particles via spark plasma sintering and hot pressing, demonstrating a significant enhancement in micro-yield strength. Through a combination of vacuum hot-pressing sintering and hot extrusion, Liu [16] et al. prepared SiCp/2024Al composites reinforced with 15 vol.% 7 μm SiC particles, attaining a tensile strength above 550 MPa following heat treatment. Although these methods can produce highly dense composites, they are often associated with high equipment costs, complex operations, and difficulties in achieving continuous production.

The study of densification in aluminum alloys and aluminum matrix composites produced by conventional powder metallurgy (cold pressing and sintering) remains an area of significant research attention. In recent decades, extensive research has been conducted on the effects of sintering aids [17,18,19], atmosphere conditions [18,20,21], and process parameters [22,23] on the densification process in aluminum powder metallurgy. Min Chul Oh [24] et al. investigated the effect of Mg content on the properties of powder metallurgy Al-Cu-Mg alloys and found that the addition of magnesium destroys the oxide film on the surface of aluminum powder, leading to the formation of a MgAl_2_O_4_ spinel structure which promotes interparticle diffusion. Du [22] et al. prepared Al-Cu-Mg alloys from elemental powders using cold pressing and pressureless sintering, investigating the sintering densification process, and their research demonstrated that optimal mechanical properties (tensile strength of 237.9 MPa) were achieved after isothermal sintering at 620 °C for 120 min. When the sintering duration was extended to 180 min, significant pore formation was observed within the material. Based on a machine learning study of copper’s role in sintering densification, Yang [25] et al. developed a SiC_p_/Al-Cu-Mg composite that attained 349 MPa in the as-sintered condition and 561 MPa after hot pressing and heat treatment. However, the aforementioned studies have primarily been conducted using elemental powders as raw materials.

While numerous studies address the fabrication of SiC_p_/Al composites, relatively fewer investigations have systematically explored the densification process of SiC_p_/Al composites fabricated from 2024 aluminum alloy powder via conventional powder metallurgy. This study employed a two-stage approach which combines sintering and hot-pressing technique to achieve densification of the SiC_p_/Al composites. The effects of sintering temperature and holding time on the densification mechanism were systematically investigated. Notably, the incorporation of pure aluminum powder into the matrix alloy powder effectively improved the compressibility of the 2024 aluminum alloy powder, laying a foundation for the cost-effective manufacturing of SiC_p_/2024 aluminum matrix composites.

## 2. Experimental Procedure

### 2.1. Materials

**Figure 1:**
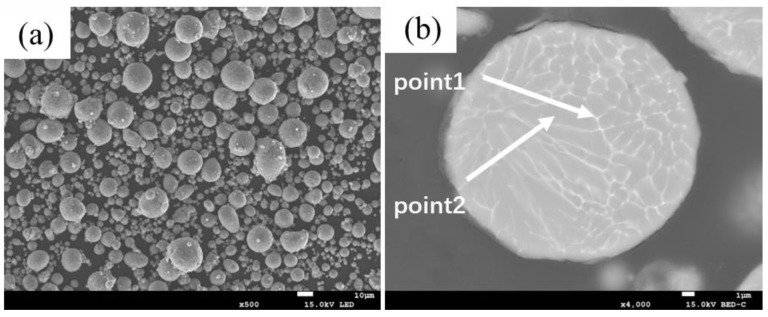
Backscattered Electron Images (BEI) of the 2024 Aluminum Alloy Particles: (**a**) Particles; (**b**) Cross-Section of a 2024 Aluminum Alloy Particle.

Gas-atomized 2024 aluminum alloy powder (median particle size of 14.1 μm), nitrogen-atomized aluminum powder (D50 = 19 μm), and silicon carbide (SiC) particles with two distinct size distributions (D50 = 31.9 μm and 66.8 μm) were used as raw materials. The matrix powder of the SiC_p_/Al composites consists of 2024 aluminum alloy powder and pure aluminum powder in a 4:1 ratio. The SiC reinforcement was incorporated at three different mass fractions (35, 40 and 45 wt.%) to investigate the effect of ceramic fraction. Figure 1 and Figure S1 illustrate the backscattered electron images (BEI) of the as-received 2024 Al alloy powder and the two grades of SiC particles, respectively. The nominal chemical composition of the 2024 aluminum alloy powder was determined using an M5000 full-spectrum direct reading spectrometer (FPI), with the results presented in Table S1. Table 1 provides the energy-dispersive X-ray spectroscopy (EDS) analysis results of the 2024 powder’s cross-sectional composition. Table 2 summarizes the designed composite formulations, including SiC content (35–45 wt.%), SiC particle size and the theoretical density for each composition.

### 2.2. Material Fabrication

The composites were fabricated through a powder metallurgy approach involving powder mixing, cold pressing, sintering, and hot pressing. The 2024 aluminum alloy powder, pure aluminum powder, and SiC particles were first uniformly blended using a single-cone mixer at a rotational speed of 90 rpm for 1 h. The mixed powders were then loaded into a steel die and compacted under a uniaxial pressure of 200 MPa with a holding time of 10 s to form green compacts. A consistent powder charge of 10 g was used for each sample. The powder compaction die and sample dimensions are shown in Figure S2. Subsequent sintering was carried out in a tube furnace under nitrogen atmosphere at varying temperatures with different holding times, followed by furnace cooling to room temperature. To further enhance densification, the sintered compacts were subjected to hot pressing in a muffle furnace, where the samples and die were co-heated to target temperatures before applying a pressure of 200 MPa for 10 s. The sample surface temperature was monitored in situ during the heating stage by inserting a fine thermocouple wire through the die gap.

### 2.3. Characterization

The differential scanning calorimetry (DSC) on the green compact sample pressed from aluminum matrix powder was analyzed using NETZSCH STA 449F5 simultaneous thermal analyzer with a nitrogen atmosphere and a heating rate of 10 °C/min up to 700 °C.

The sintering shrinkage rate of the samples was calculated according to Equation (1):

(1) $$A=\left(L-L_{0}\right)/L_{0}$$

where L represents the sample length after sintering and $L_{0}$ denotes the sample length before sintering.

The real density of the samples was measured using the Archimedes drainage method and calculated according to Equation (2):

(2) $$\rho_{s}=\frac{\rho_{w}W_{g}}{W_{g}W_{w}}$$

where $\rho_{s}$ represents the real density of the sample (g/cm^3^), $\rho_{w}$ denotes the density of water (g/cm^3^), $W_{g}$ is the mass of the sample in air (g), and $W_{w}$ indicates the mass of the sample in water (g). Since the sintered samples contained certain pores, a very thin layer of vaseline was uniformly applied to the sample surface before testing. The relative density at each processing stage (green, sintered, hot-pressed) is calculated as the percentage of the measured density to the theoretical density of the material.

The phase composition of the composite material was characterized using a SmartLab SE fully automatic multifunctional X-ray diffractometer (Rigaku Corporation, Tokyo, Japan) equipped with an XSPA400 2D detector under Cu Kα radiation. The tensile strength of composites was tested using an AGS-X-100KN universal testing machine (Shimadzu Corporation, Kyoto, Japan) with the average value of four specimens taken for each test group. The metallographic microstructure was observed using a Zeiss Axiolab 5 optical microscope (Carl Zeiss Microscopy GmbH, Jena, Germany). Microstructure, energy dispersive spectroscopy (EDS), and fracture morphology were characterized using a Tescan Mira 4 field emission scanning electron microscope (TESCAN, Brno, Czech Republic) equipped with an Oxford Xplore 30 EDS system (Aztec one software module version 6.2, Oxford Instruments, High Wycombe, UK). Samples for microstructural analysis were extracted from the ends of the tensile specimens and were polished with emery papers of varying grades, followed by a final polish using diamond suspension.

## 3. Results and Discussion

### 3.1. Sintering Temperature

Figure S3 shows the DSC curve of a green compact pressed from the matrix powder of SiC_p_/Al-1 composite material, measured under high-purity nitrogen atmosphere. Only one distinct endothermic peak is visible on the curve at 653 °C. This shows a notable difference from the DSC curve of the Al-Cu-Mg powder compact prepared from elemental powders, where distinct endothermic peaks representing the formation of eutectic liquid phase can be observed [22]. Based on the DSC results, the study investigated the material evolution of SiC_p_/Al-1 composite at different sintering temperatures within the range of 460–640 °C to clarify its sintering densification process.

**Figure 2:**
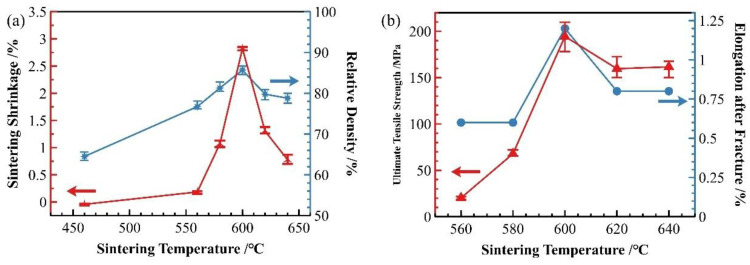
Effect of sintering temperature on properties of SiC_p_/Al-1: (**a**) sintering shrinkage rate and sintered relative density; (**b**) strength and elongation rate of sintered samples.

Figure 2 presents the sintering shrinkage rate, relative density, and ultimate tensile strength of SiC_p_/Al-1 composite samples sintered for 30 min at different temperatures. The shrinkage rate, relative density, strength, and elongation after fracture of the samples all initially increased and then decreased as the temperature increased.

**Figure 3:**
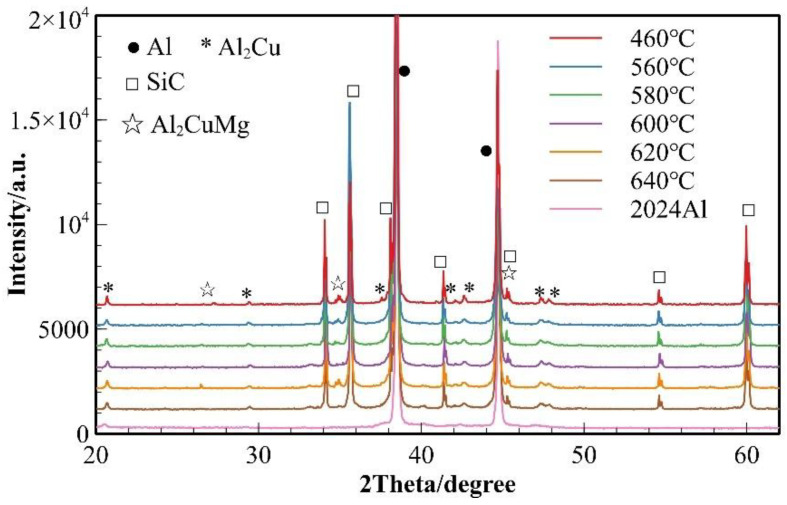
XRD patterns of SiC_p_/Al-1 samples held at different sintering temperatures for 30 min.

To investigate the effect of sintering temperature on the phase evolution of the composite material, XRD phase analysis was performed on 2024 aluminum alloy powder and SiC_p_/Al-1 composites prepared at various sintering temperatures (Figure 3). Only the diffraction peaks of α (Al) were observed in the 2024 aluminum alloy powder. The phase composition of the composites remained essentially consistent across all sintering temperatures, consisting of α (Al) matrix, θ (Al_2_Cu) and S (Al_2_CuMg) phases, and SiC reinforcement. For the sample sintered at 460 °C, the α (Al) peaks showed the highest intensity. Meanwhile, it was observed that the diffraction peaks of θ (Al_2_Cu) phase exhibited a gradual broadening trend with increasing temperature, which was analyzed using Scherrer’s equation (Equation (3)):

(3) $$D=\frac{k\lambda }{\beta cos\theta }$$

where D represents the average grain size (typically perpendicular to the diffracting crystal planes) with units of nanometers (nm); k is the Scherrer constant (shape factor), which is dimensionless; λ denotes the wavelength of the X-rays; β corresponds to the full width at half maximum (FWHM) of the diffraction peak; and θ is the Bragg angle of the diffraction peak. The equation demonstrates that larger FWHM values of diffraction peaks correspond to smaller grain sizes. Therefore, it can be preliminarily inferred that the grain size or crystallinity of the θ phase (Al_2_Cu) undergoes corresponding changes with variations in sintering temperature.

**Figure 4:**
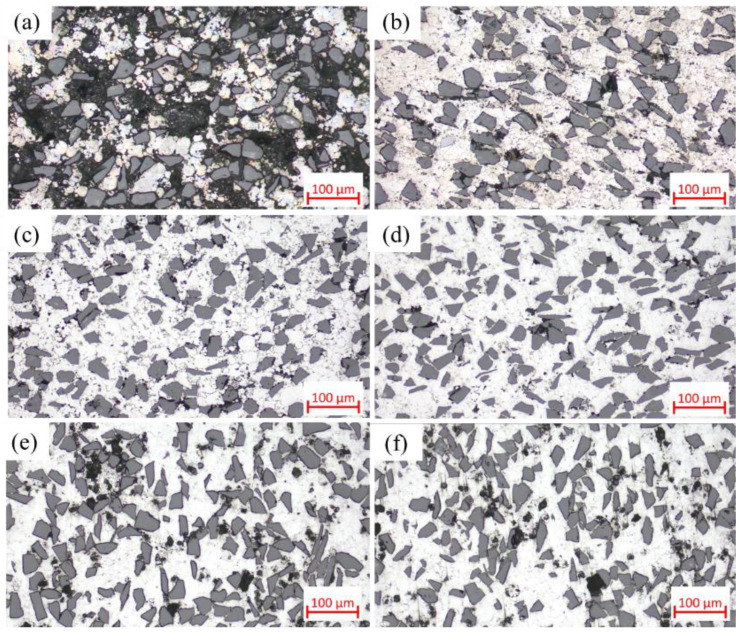
Optical micrographs of SiC_p_/Al-1 samples held at different sintering temperatures for 30 min: (**a**) 460 °C; (**b**) 560 °C; (**c**) 580 °C; (**d**) 600 °C; (**e**) 620 °C; (**f**) 640 °C.

Figure 4 shows the optical micrographs of SiC_p_/Al-1 composites prepared at different sintering temperatures, which exhibits significant microstructural differences. In the sample sintered at 460 °C, most matrix powders and SiC particles fail to establish effective metallurgical bonding, primarily relying on mechanical interlocking through matrix deformation. The weak interfacial bonding leads to particle detachment during sample preparation, resulting in observable material loss and distinct interparticle voids in the micrographs. With increasing sintering temperature, the composite’s densification progressively improves, accompanied by enhanced interparticle bonding. However, when the temperature reaches 620 and 640 °C, noticeable voids reappear in the microstructure—a phenomenon consistent with the observed relative density variation trends.

**Figure 5:**
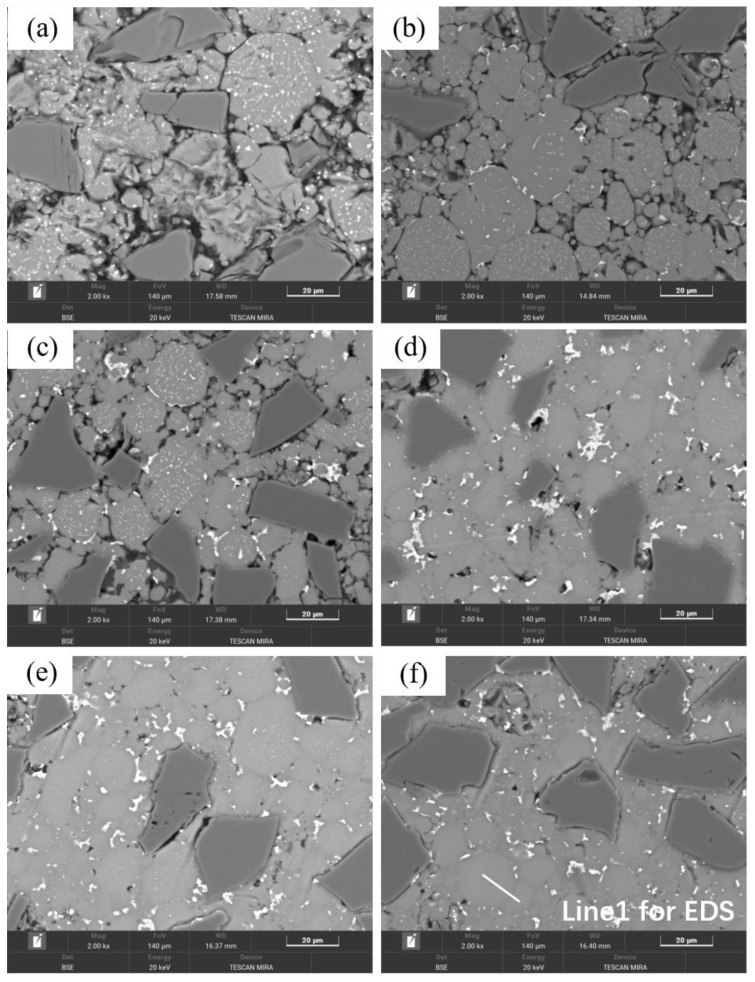
Backscattered electron images (BEI) of SiC_p_/Al-1 samples held at different sintering temperatures for 30 min: (**a**) 460 °C; (**b**) 560 °C; (**c**) 580 °C; (**d**) 600 °C; (**e**) 620 °C; (**f**) 640 °C.

**Figure 6:**
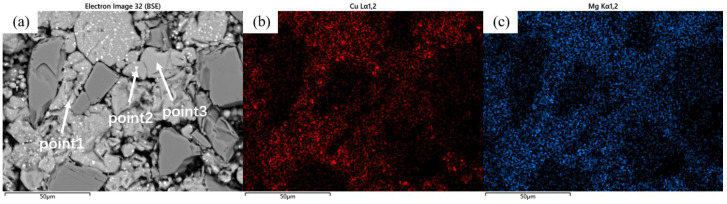
(**a**) Backscattered electron image (BEI) of SiC_p_/Al-1 sample after heat preservation at 460 °C for 30 min; corresponding EDS spectra of (**b**) Cu and (**c**) Mg.

As shown in Figure 5, in addition to the degree of densification, the precipitates in the material’s microstructure evolved significantly with sintering temperature. As the temperature increased, the precipitation sites progressively transitioned from the particle interiors to the grain boundaries. For the samples sintered at 460 °C, distinct dot-like precipitates were observed within or on the surface of most matrix particles. EDS analysis (Figure 6) of the sample revealed that the distribution of Cu elements generally coincided with the bright spots in the BEI, while certain regions showed significantly lower Cu content, corresponding to the pure aluminum powder particles. As a primary alloying element, the presence of Mg element exhibited a more uniform distribution compared to Cu, with their enrichment zones not completely overlapping.

Combined with the EDS results from Table 3, two main precipitate phases were identified in the 460 °C-sintered samples: an Al-Cu-Mg-Si phase and an Al-Cu phase. For the Al-Cu phase, the Al-Cu mass ratio is greater than that in the Al_2_Cu phase. For the Al-Cu-Mg-Si phase, Point 1 in Table 3, located near the particle surface, exhibited notably higher oxygen content compared to other measurement points. Previous studies on the sintering densification of powder metallurgy aluminum-based materials suggest that Mg participates in breaking the oxide layer on Al or Al alloy powder surfaces, forming Al-Mg-O compounds [17,23]. According to the Al-Mg binary phase diagram, a eutectic reaction occurs at 450 °C. When elemental powder is used to prepare powder metallurgy aluminum alloys or composites, an Al-Mg eutectic liquid phase forms at this temperature, causing rapid diffusion of Mg to the Al powder surface where it reacts with the oxide layer. The formation of this eutectic liquid phase and elemental diffusion leads to the disappearance of original Mg-rich particles, creating numerous voids. In this study, as shown in Figure 1 and Table 1, in 2024 aluminum alloy powder, the primary initial forms of Cu and Mg are solid solutions in the α (Al) matrix and divorced eutectic structures formed at the grain boundaries within the powder particles [26]. As temperature increased, the original grain boundaries within the 2024 powder particles disappeared, the initially segregated Cu and Mg at grain boundaries diffused into the α (Al) matrix, and the divorced eutectic structure gradually decomposed into S-phase (Al_2_CuMg) and θ-phase (Al_2_Cu). Concurrently, Mg reacted with the oxide layer on the aluminum alloy powder surface and with silicon or silica residue retained on the SiC particles, locally forming multiphase regions consisting of Mg_2_Si + Al_2_CuMg/Al_2_Cu + MgAl_2_O_4_. Due to the low concentration of Mg_2_Si and MgAl_2_O_4_, these phases were not detected in the XRD analysis. Based on the EDS results and the XRD patterns in Figure 3, indicating that the precipitated phases are Al-Cu and Al-Cu-Mg intermediate phases with higher aluminum content than those of the θ-phase and S-phase.

Besides changes in the 2024 Al alloy powder, the pure Al powder particles also underwent transformations. Due to elemental diffusion, Cu, Mg, Fe, and Si were detected within these particles, with Fe and Si contents significantly higher than in the 2024 alloy powder. This indicates that solid-state diffusion has occurred between the 2024 aluminum alloy powder and the pure aluminum powder. The anomaly in Fe content may originate from the mixing process or impurities in the pure aluminum powder, while for Si content may derive from free silicon or silicon dioxide residues on the silicon carbide particles. Mn, with its slower diffusion rate, was not detected at this temperature.

Furthermore, as shown in Figure 2, samples sintered at 460 °C exhibited slight expansion, consistent with densification studies of powder metallurgy Al alloys. At this temperature, the material’s density decreased slightly compared to the as-pressed state due to thermal expansion and stress relaxation in the deformed Al matrix particles [27]. Unlike systems using pure Al powder, where particles tend to spheroidize at this temperature [23], the Al and Al alloy powder here retained clear evidence of plastic deformation from pressing, with straight particle boundaries and no significant spheroidization.

**Figure 7:**
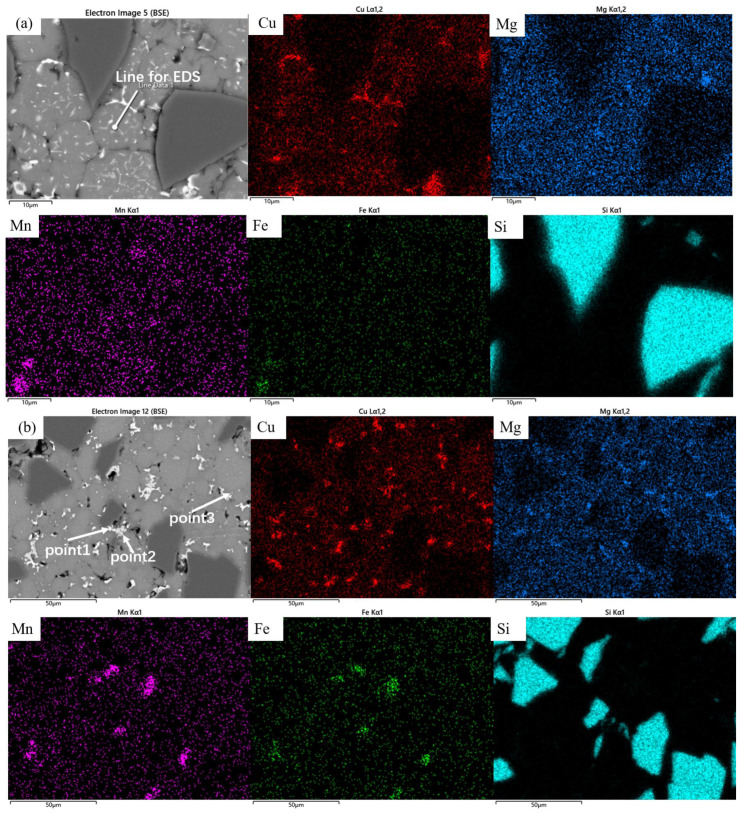
Backscattered electron image (BEI) of the SiCp/Al-1 sample after holding at (**a**) 560 °C and (**b**) 600 °C for 30 min, along with their corresponding EDS elemental maps showing the distribution of Cu, Mg, Mn, Fe, and Si.

**Figure 8:**
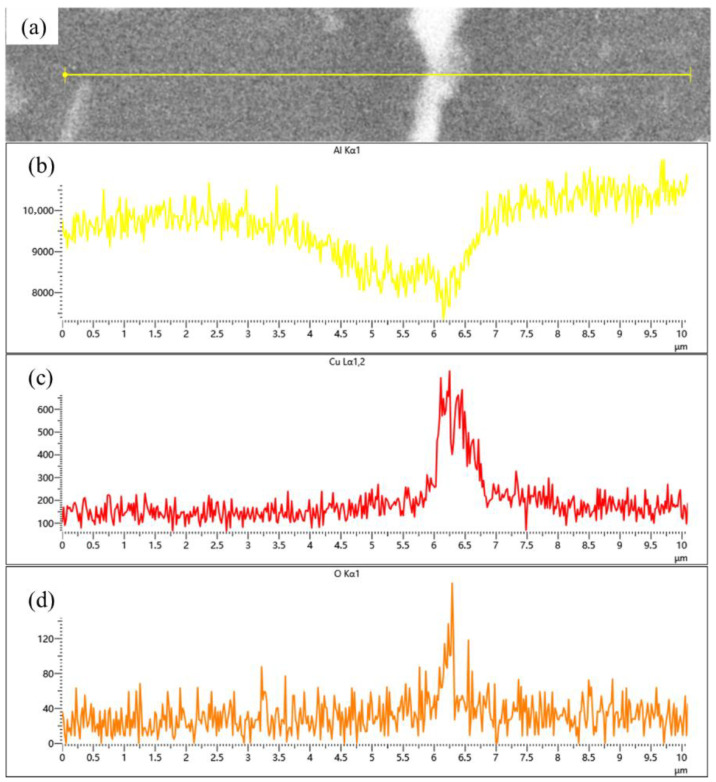
(**a**) Backscattered electron image (BEI) of the particle interface in SiC_p_/Al-1 after holding at 560 °C for 30 min; corresponding EDS spectra of (**b**) Al, (**c**) Cu, and (**d**) O, respectively.

The samples sintered at 560 °C demonstrated significantly enhanced densification compared to those processed at 460 °C, with evident interparticle bonding formation. Compared to the coarse dot-like precipitates observed within the matrix particles at a sintering temperature of 460 °C, distinct needle-like and fine dot-like precipitates have now formed inside the particles, with the size of the dot-like phases significantly reduced. The previously plastically deformed matrix particles exhibited noticeable spheroidization transformation. However, distinct interfaces and voids remained observable, primarily categorized into two types: interfacial voids at ceramic particle/matrix boundaries and triangular voids formed between matrix particles. At the same time, the precipitation morphology underwent substantial transformation, exhibiting continuous precipitates along particle edges (Figure 7a). Elemental distribution analysis revealed more uniform dispersion of Cu and Mg compared to the low-temperature condition, with Cu predominantly enriched in the continuous intergranular precipitates. EDS line scanning confirmed these grain-boundary precipitates as Al-Cu compounds (Figure 8), while slightly elevated oxygen content was detected at particle surfaces. According to the Al-Cu binary phase diagram, these interfacial precipitates were identified as Al-Cu eutectic liquid phase. Additionally, Mn- and Fe-enriched precipitates were observed among particles alongside the Al-Cu eutectic phases.

As shown in Figure 2a, the relative density of the material sintered at 560 °C significantly increased due to the formation of abundant Al-Cu eutectic liquid phase at this temperature. The liquid phase precipitates on particle surfaces and preferentially fills interparticle gaps through capillary action. During this process of liquid phase filling and flow, simultaneous particle rearrangement occurs, leading to enhanced material densification. However, as evident in Figure 2b, the material still exhibited relatively low strength because of clearly visible interparticle interfaces and relatively weak bonding at this stage.

As shown in Figure 5c, for samples sintered at 580 °C, the precipitation characteristics at particle edges and interiors remained similar to those observed at 560 °C. The increased temperature further promoted the precipitation of eutectic liquid phase, making the interparticle precipitates more distinct and consequently enhancing material densification. Compared to the 560 °C condition, the intragranular precipitates within the matrix particles showed some degree of coarsening. While well-defined sintering necks formed between particles, a number of voids persisted both among matrix particles and at the interfaces between matrix and ceramic particles.

The samples sintered at 600 °C showed significant improvement in densification. As shown in Figure 2, both the relative density and mechanical properties of the samples sintered at this temperature were markedly enhanced. The microstructure also underwent substantial changes (Figure 7b), with the number of voids significantly reduced—the remaining voids being primarily near-spherical “triangular zone” voids between matrix particles. The precipitates at matrix grain boundaries increased, and the bonding between matrix particles became more compact, exhibiting distinct gray boundaries in the images. Meanwhile, both the size and quantity of intragranular precipitates significantly decreased, which is consistent with the peak broadening analysis results shown in Figure 3. At this temperature, excellent bonding between silicon carbide particles and the matrix was observed. Elemental mapping analysis revealed partial overlap in the distribution of Cu and Mg, while Mn and Fe showed highly coincident distribution patterns. Combined SEM-EDS analysis (Figure 7b and Table 4) identified three main types of interparticle precipitates in 600 °C-sintered samples: (1) bright white Al-Cu precipitates; (2) coarse gray Al-Mn-Fe-Si precipitates, which is determined to be the (Fe,Mn,Si)Al_6_ phase; and (3) finer gray Al-Cu-Mg-Si-O precipitates [28]. Compared to low-temperature sintered samples, the Mn- and Fe-containing precipitates increased substantially. The elevated temperature promoted greater liquid phase formation, while reduced surface tension enhanced the wettability of the liquid phase on matrix particle surfaces, thereby improving void-filling effectiveness.

When the sintering temperature was further increased to 620 °C, significant changes occurred in the material’s characteristics. As shown in Figure 2, both the relative density and mechanical properties of samples sintered at 620 °C decreased, and this declining trend persisted at 640 °C. The microstructure of 640 °C-sintered samples closely resembled that of the 620 °C specimens. Figure 4 reveals that compared to 600 °C samples, those sintered at 620 and 640 °C developed notably coarser pores. According to Figure S3, at these elevated temperatures, substantial liquid phase formation, combined with thermal expansion, hindered gas escape and led to pore formation.

As observed in Figure 5, the precipitates in 620 and 640 °C samples remained similar in morphology to those at 600 °C. Notably, interfacial gaps between SiC particles and the matrix reappeared, resembling those in low-temperature sintered samples, indicating altered interfacial bonding characteristics. This phenomenon can be attributed to two factors: (1) the more pronounced thermal expansion coefficient mismatch between the aluminum matrix and SiC particles at high temperatures, led to interfacial debonding during cooling; and (2) enhanced interfacial reactions formed detrimental Al_4_C_3_ phases that weakened bonding strength. As shown in Figure 4d–f and Figure 5d–f, while smaller-sized voids decreased, larger pores emerged with noticeable void coarsening. This phenomenon occurs because thermal energy causes grain boundaries to move faster than pores can migrate, resulting in the separation of grain boundaries from pores and the formation of isolated residual pores [29].

**Figure 9:**
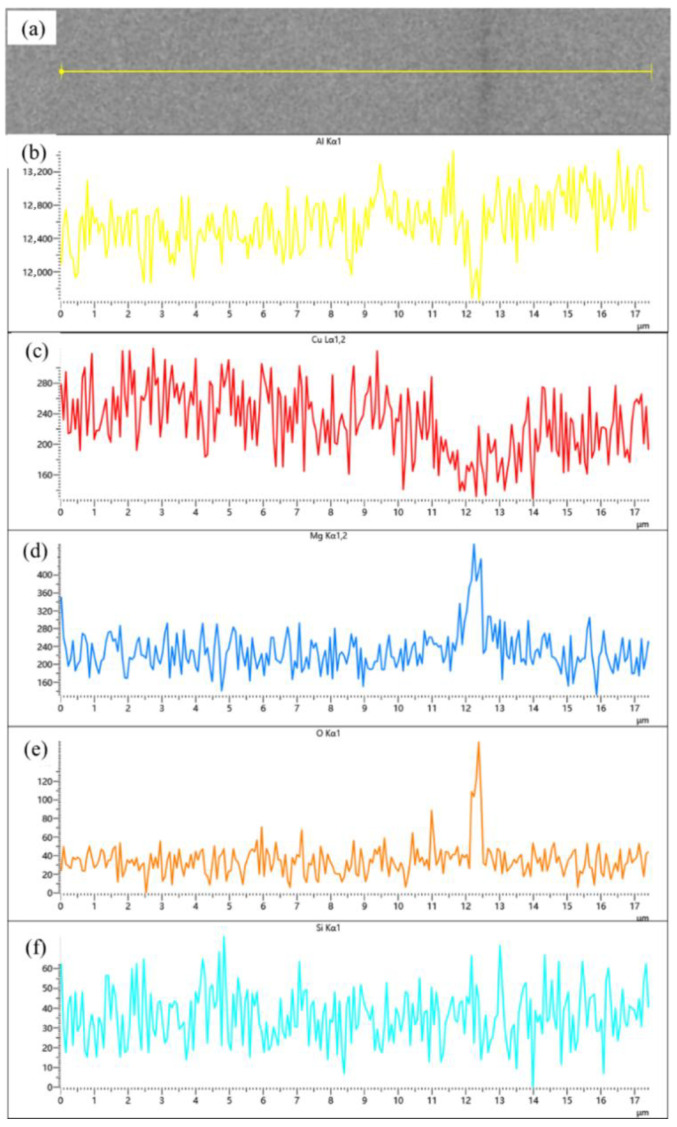
(**a**) Backscattered electron image (BEI) of the particle interface in SiC_p_/Al-1 after holding at 640 °C for 30 min; corresponding EDS spectra of (**b**) Al, (**c**) Cu, (**d**) Mg, (**e**) O, and (**f**) Si, respectively.

Compared samples sintered at 600, 620 and 640 °C, a significant improvement in material relative density is observed compared to low-temperature sintering conditions. Notably, a distinct gray boundary with measurable width forms between matrix particles, through which the original particle morphology remains discernible. Figure 9 presents localized microstructural features and elemental distribution profiles across an interparticle boundary in a 640 °C-sintered sample. The boundary region, measuring approximately 2–4 μm in width, exhibits marked elemental segregation: Mg and O concentrations are significantly elevated at the boundary compared to particle interiors, while Al and Cu contents show relative depletion. Silicon distribution, however, remains essentially uniform without noticeable variation between the boundary and adjacent regions. According to the energy spectrum results, the observed gray boundaries exhibit enrichment of Mg and O elements. It is inferred that the primary components originate from aluminum oxide on the surface of aluminum powder and aluminum alloy powder, as well as MgO or MgAl_2_O_4_ formed by the reaction between magnesium and the oxide layer. This finding further confirms the disruptive effect of magnesium elements on the oxide layer. This phenomenon has also been observed in aluminum alloys and aluminum matrix composites prepared by powder metallurgy methods. A. Kimura et al. [30] found that magnesium in the alloy powder migrates from the interior to the surface and reduces the aluminum oxide on the powder surface, in 55 vol.% SiC_p_/6061 Al composites fabricated via semi-solid hot isostatic pressing by Kun Cheng et al. [10], a continuous 5–10 nm thick Al-O-Mg interfacial layer was observed between the SiC_p_ and the 6061 Al matrix.

Based on the variations in relative density, mechanical properties, phase composition, and microstructure at different sintering temperatures, the temperature-dependent sintering process of SiC_p_/Al-1 composites can be divided into three distinct stages.

Stage I (Room Temperature to 460 °C):

Compared to the original microstructure of the 2024 aluminum alloy powder (Figure 1b), which consists of fine grains and divorced eutectics at grain boundaries, the divorced eutectics at the original particle boundaries gradually decompose into α-Al, θ-phase (Al_2_Cu) and S-phase (Al_2_CuMg) as temperature increases. During this stage, the densification of the composite progresses slowly.

Stage II (460 °C to 580 °C):

According to the Al-Cu binary phase diagram [31], as the temperature further rises into the α-Al single-phase region, the θ-phase dissolves into α-Al, causing the grain boundaries within the 2024 aluminum alloy particles to disappear, and α-Al grains merge such that one original particle forms a single grain. Due to the rapid heating rate, the temperature quickly reaches or exceeds the eutectic point before the θ-phase can fully dissolve into α-Al, leading to melting. To minimize solid/liquid interfacial energy, the liquid phase migrates toward particle boundaries. Since the liquid phase originates from θ-phase melting and is rich in Cu, it diffuses into and dissolves within the α-Al of surrounding pure aluminum particles. The precipitation of the eutectic liquid phase promotes particle rearrangement and grain growth, enhancing the composite’s density and imparting moderate strength. Concurrently, the eutectic liquid phase precipitation is accompanied by the segregation of elements such as Mn and Fe at grain boundaries.

Stage III (580 °C to 600 °C):

At this stage, sufficient eutectic liquid phase forms, and the high temperature improves the wettability of the liquid phase on particle surfaces, significantly increasing the material’s density and mechanical properties. The precipitation phases rich in Mn, Fe, and other elements become notably more abundant. Effective bonding is established between SiC particles and aluminum matrix particles, as well as between aluminum matrix particles themselves, with distinct Mg- and O-rich boundaries forming at the interfaces.

Stage IV (600 °C to 640 °C):

As the sintering temperature is further increased beyond 600 °C, pore coarsening and interfacial weakening occur, which are detrimental to material densification.

### 3.2. Holding Time

**Figure 10:**
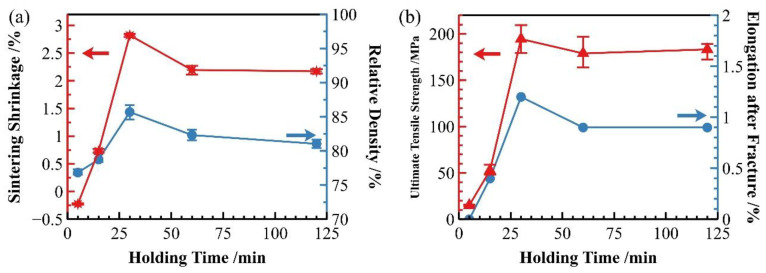
Properties of as-sintered SiC_p_/Al-1 at 600 °C with different holding times: (**a**) sintering shrinkage rate and sintered relative density; (**b**) strength and elongation of sintered samples.

In addition to sintering temperature, holding time also significantly influences the sintering densification process of the material. As discussed in Section 3.1 regarding sintering temperature, 600 °C has been identified as the optimal sintering temperature. At this temperature, we investigated the effect of different holding times on the sintering densification of SiC_p_/Al-1 composites. As shown in Figure 10a, when sintered at 600 °C, both the sintering shrinkage rate and relative density of the samples increased rapidly with prolonged holding time, reaching peak values at 30 min. Beyond 30 min, the shrinkage rate showed a declining trend before gradually stabilizing. Correspondingly, the relative density of sintered samples followed essentially the same trend as the shrinkage rate.

Figure 10b demonstrates that the strength of sintered samples exhibits a clear correlation with their densification degree. Both the strength and elongation of sintered samples follow trends similar to the shrinkage rate. When holding time was extended from 15 to 30 min, the material’s strength showed rapid improvement, peaking at 30 min. Further extension of holding time resulted in a slight decrease in strength, though it remained substantially higher than values obtained at 5 and 15 min holding times.

**Figure 11:**
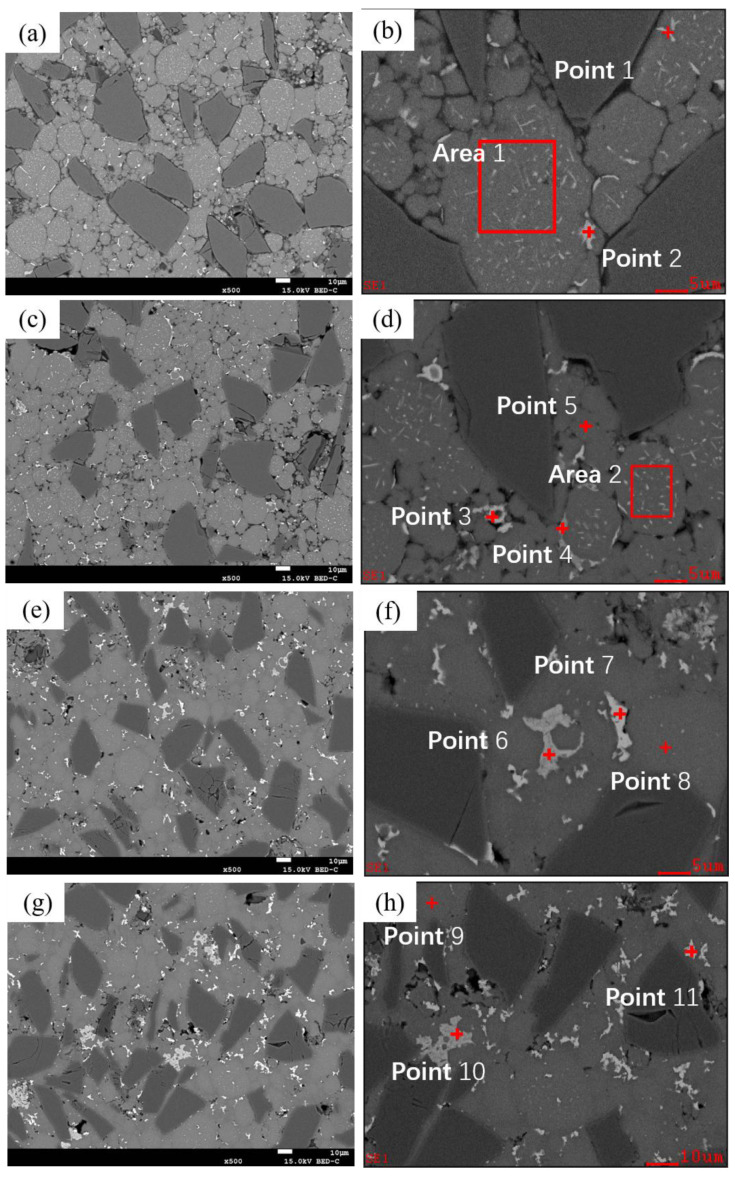
Backscattered Electron Images (BEI) of as-sintered SiC_p_/Al-1 samples at different holding times under a sintering temperature of 600 °C: (**a**,**b**) 5 min; (**c**,**d**) 15 min; (**e**,**f**) 60 min; (**g**,**h**) 120 min.

As shown in Figure 5d and Figure 11, the microstructure of as-sintered SiC_p_/Al-1 exhibits significant differences under various holding times at a sintering temperature of 600 °C.

When the holding time is 5 min, the boundaries between the matrix particles remain distinct. Sintering necks can be seen forming between a few particles. Within the matrix particles, two types of particles are observed: one with noticeable dot-like and needle-like precipitates inside, and the other without obvious precipitates. Based on the compositional design, these two types correspond to 2024 aluminum alloy powder particles and pure aluminum powder particles, respectively. A small number of precipitates are observed along the edges of some matrix particles. According to EDS results (Table 5), the precipitates are primarily composed of Al-Cu, with the Cu content close to the eutectic composition of Al-Cu. Due to the precipitation of the liquid phase, the Cu content inside the 2024 alloy powder particles is lower than the theoretical value compared to Mg. The short processing cycle resulted in rapid solidification during powder preparation, Mn and Fe elements mainly exist as supersaturated solid solutions or nano-sized precipitates, making them undetectable by EDS at this stage.

When the holding time is extended to 15 min, more sintering necks form between particles. Dot-like and needle-like precipitates are still observed within the matrix particles, while precipitates along the particle edges significantly increase. In addition to the Al-Cu eutectic liquid phase, Al-Mn-Fe phase precipitates are also detected at the particle boundaries. At this point, Mn and Fe elements can be identified inside the alloy powder particles (e.g., Point 5).

At a holding time of 30 min, the precipitates within the grains noticeably decrease, while those at the particle boundaries continue to increase, and the particle boundaries become less distinct. After 60 min of holding, similar to the 30 min condition, intragranular precipitates further diminish, and more precipitates accumulate in the triangular regions between particles, beginning to coarsen. The Al-Mn-Fe-Si and Al-Cu phases exhibit dark gray and light gray colors, respectively. Based on energy spectrum analysis, the Al-Mn-Fe-Si phase is identified as (Fe,Mn,Si) Al_6_, while the Al-Cu phase corresponds to the θ phase (Al_2_Cu). Additionally, the Mg content in the matrix aligns with the theoretical value, while the Cu content is slightly lower due to the presence of θ phase at particle boundaries.

When the holding time is further extended to 120 min, the interparticle (Fe,Mn,Si) Al_6_ and θ phases significantly increase and coarsen. As time progresses, the bonding between particles becomes tighter, voids gradually decrease and become more rounded, and coarse precipitates within the matrix particles disappear. Meanwhile, interparticle precipitates continue to grow and coarsen.

### 3.3. Hot-Pressing Process

As analyzed in the previous section regarding the sintering densification behavior of SiC_p_/Al-1 composite materials under different sintering processes, achieving full densification during the sintering stage proved to be challenging. To further investigate the performance potential of SiC_p_/Al-1 composites through subsequent hot deformation for additional densification, hot pressing was conducted on sintered samples at varying temperatures.

The sintered samples were obtained by sintering SiC_p_/Al-1 composites at 600 °C for 30 min. Figure S4 shows the samples after hot pressing at different temperatures. As the temperature increased, the material’s deformation resistance decreased, which was evidenced by the flash formation along the sample edges.

**Figure 12:**
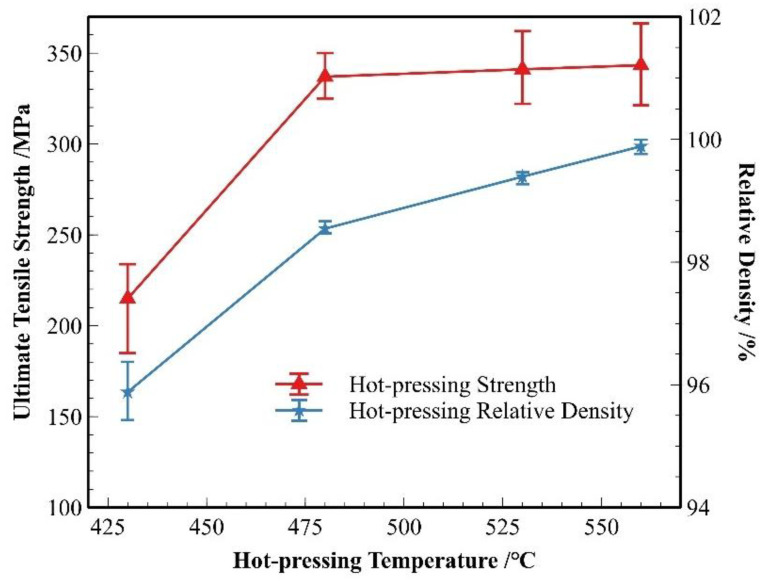
Strength and density of SiC_p_/Al-1 after hot pressing at different temperatures.

Figure 12 presents the strength and relative density data of the material after hot pressing at different temperatures. As shown in the figure, both the strength and relative density of the material significantly improved after hot pressing. The most notable enhancement in material strength and densification occurred as the hot-pressing temperature increased from 430 °C to 480 °C. With further increases in temperature, the change in relative density became more pronounced compared to the change in strength.

**Figure 13:**
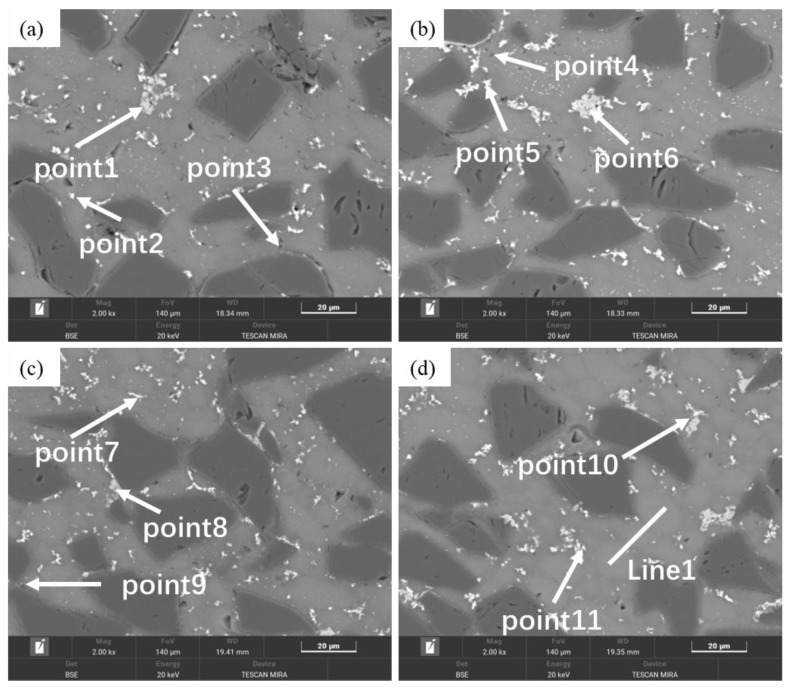
Backscattered electron images (BEI) of SiC_p_/Al-1 after hot pressing at different temperatures: (**a**) 430 °C; (**b**) 480 °C; (**c**) 530 °C; (**d**) 560 °C.

**Figure 14:**
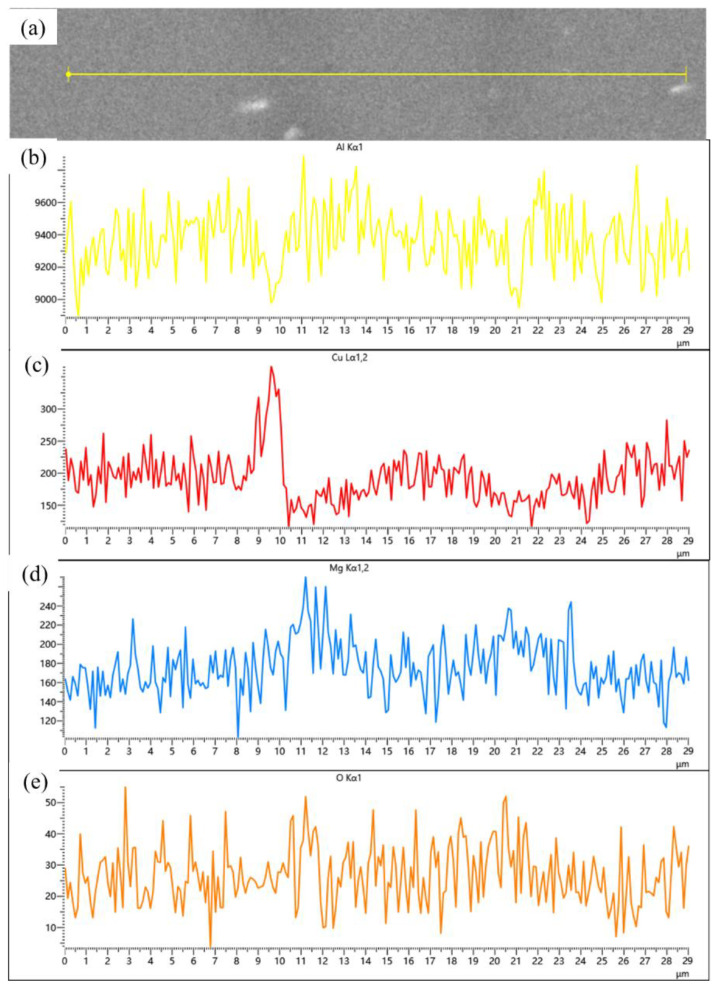
(**a**) Backscattered electron image (BEI) of the particle interface in SiC_p_/Al-1 after hot pressing at 560 °C; corresponding EDS spectra of (**b**) Al, (**c**) Cu, (**d**) Mg, and (**e**) O, respectively.

Figure 13 shows the microstructure of SiC_p_/Al-1 after hot pressing at different temperatures. It can be observed that the hot-pressing process significantly improved the material’s densification. Only a small number of micropores were detected in the matrix of the sample hot-pressed at 430 °C, while no obvious pores were found in samples processed at other temperatures. Compared to the as-sintered state (Figure 5d), the hot-pressed samples exhibited slightly reduced and more uniformly distributed precipitate phases within the aluminum alloy matrix, particularly under hot-pressing conditions of 530 and 560 °C. Combined with SEM and EDS data (Table 6), the precipitates in the hot-pressed samples mainly consisted of three types: Al-Fe-Mn-Si, Al-Cu, and Al-Mg-Si-O, with their sizes decreasing in that order. As shown in Figure 14, the gray Mg- and O-rich interparticle boundaries observed in the sintered microstructure remain visible in the hot-pressed state. In the hot-pressed condition, the concentration gradients of Mg and O elements at these interparticle boundaries decrease gradually toward the particle interiors, indicating that hot pressing promotes a more homogeneous distribution of Mg and O. Additionally, fine needle-like and dot-shaped precipitates can be observed within the matrix particles, which are presumed to correspond to the θ-phase and S-phase.

During the hot-pressing process, the rising temperature promotes continuous dissolution of precipitate phases such as θ phase (Al_2_Cu) into the α-Al matrix. Consequently, at the three temperatures of 430, 480 and 530 °C, the precipitates in the 530 °C sample appear the finest and most uniform. However, since no holding treatment was applied at any hot-pressing temperature, the dissolution may be incomplete. Additionally, as the samples were cooled in the mold rather than quenched after hot pressing, even processing at 530 °C did not achieve a solid solution effect, preventing complete homogeneous distribution of Cu elements. When the hot-pressing temperature reaches 560 °C, eutectic liquid phases form again, leading to a relative increase in precipitates compared to samples hot-pressed at 530 °C.

**Figure 15:**
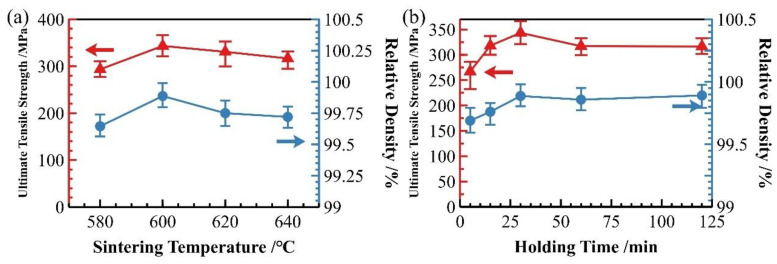
Relative density and strength of SiCp/Al-1 composites after hot-press densification under different sintering conditions: (**a**) varying sintering temperatures; (**b**) varying holding times.

From the perspective of relative density and strength variations after hot pressing, the hot-pressing process significantly enhances both the densification and mechanical properties of as-sintered aluminum matrix composites, serving as an effective method to improve overall material performance. Figure 15 presents the relative density and strength data of samples subjected to different sintering temperatures (with a 30 min holding time) and varying holding times at 600 °C after hot pressing. The results show that the trends remain largely consistent with those of the as-sintered state, with all hot-pressed samples exhibiting improved density and strength across different processing conditions.

For samples with different holding times, those sintered for less than 15 min still demonstrate significantly lower performance compared to others. As analyzed earlier regarding the influence of sintering duration, insufficient sintering time results in clearly distinguishable particle boundaries and ineffective interfacial bonding. This indicates that while hot pressing can enhance material density, it cannot fully substitute the interfacial bonding achieved during sintering. Therefore, ensuring adequate holding time during sintering is crucial for optimizing material performance.

Based on the comprehensive evaluation of relative density, mechanical properties and microstructure characteristics of both as-sintered and hot-pressed specimens, the optimal sintering parameters for SiC_p_/Al-1 composites were determined to be 600 °C with 30 min holding duration. For subsequent hot-pressing treatment, the optimal processing temperature range was identified as 530–560 °C, which resulted in superior enhancement of the material’s properties.

### 3.4. Content and Particle Size of Reinforcement Phase

The size and content of SiC particles in the SiC_p_/Al composites significantly influence both the processing characteristics and material properties. As per the compositional design in Table 3, four composite variants (SiC_p_/Al-1, SiC_p_/Al-2, SiC_p_/Al-3, and SiC_p_/Al-4) were prepared under a sintering condition of 600 °C/30 min and a hot-pressing temperature of 560 °C for systematic evaluation: SiC_p_/Al-1 and SiC_p_/Al-2 were designed for performance comparison with identical SiC content but different particle sizes; SiC_p_/Al-1, SiC_p_/Al-3, and SiC_p_/Al-4 were configured to investigate the effects of varying SiC content while maintaining a consistent particle size.

**Figure 16:**
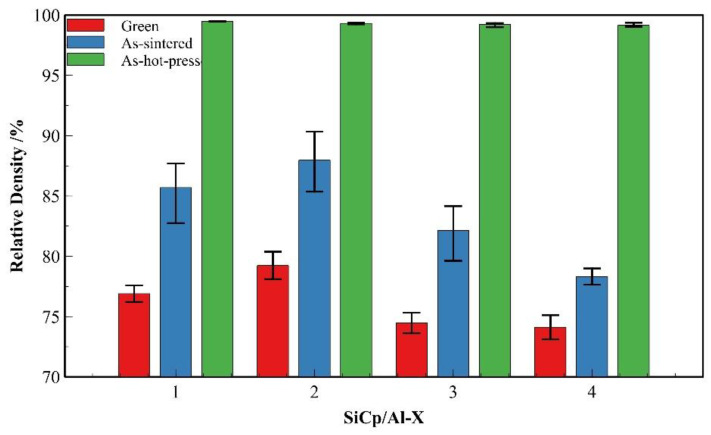
Comparison of densification at different processing stages for various SiC_p_/Al composites.

Figure 16 presents a comparative analysis of the relative densities of four SiC_p_/Al composites (SiC_p_/Al-1 to SiC_p_/Al-4) in three processing states: as-compacted, as-sintered, and hot-pressed. The comparison between SiC_p_/Al-1, SiC_p_/Al-3 and SiC_p_/Al-4 demonstrates that increasing SiC particle content leads to reduced densities in both as-compacted and as-sintered states. This phenomenon can be attributed to two primary factors: (1) higher SiC content increases the difficulty of particle plastic deformation and mechanical interlocking during powder compaction, thereby reducing powder compressibility; (2) the decreased aluminum matrix volume results in less liquid phase formation during sintering, which adversely affects particle rearrangement and subsequent densification. In contrast, the comparison between SiC_p_/Al-1 and SiC_p_/Al-2 reveals that coarser ceramic particles contribute to higher densities in both as-compacted and as-sintered states. At identical SiC mass fractions, larger particle sizes correspond to fewer SiC particles and improved powder flowability, enhancing compactibility during forming. Additionally, the reduced interfacial area between SiC particles and aluminum matrix facilitates better wetting during sintering. In the hot-pressed state, all four composites achieve near-full density. The hot-pressing process effectively compensates for the densification limitations encountered during earlier processing stages, demonstrating its crucial role in achieving optimal composite density.

**Figure 17:**
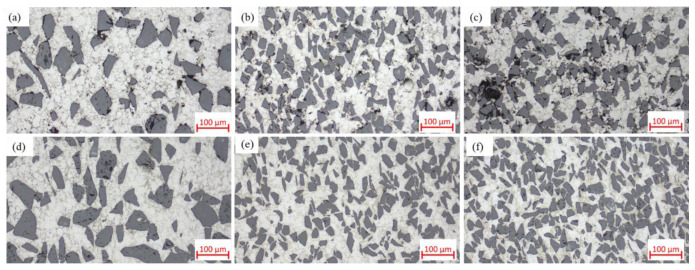
Optical micrographs of as-sintered SiC_p_/Al composites: (**a**) SiC_p_/Al-2; (**b**) SiC_p_/Al-3; (**c**) SiC_p_/Al-4; optical micrographs of hot-pressed SiC_p_/Al composites: (**d**) SiC_p_/Al-2; (**e**) SiC_p_/Al-3; (**f**) SiC_p_/Al-4.

Compare Figure 4d, Figure 14d, and Figure 17. The variations in microstructural characteristics of each composite composition are consistent with the changes in relative density. In the as-sintered state, as the SiC particle content increases, the voids in the material become larger and more numerous, primarily forming between clusters of SiC particles. Notably, in SiC_p_/Al-4, significantly larger voids enclosed by aggregated SiC particles are observed. Conversely, when the SiC particle size increases, the number of voids decreases. In the hot-pressed state, all composites exhibit nearly void-free microstructures with uniformly distributed SiC particles, showing no significant agglomeration even in high-SiC-content materials. This is attributed to the small particle size ratio between the matrix and the SiC particles [16]. These microstructural observations are consistent with the relative density variations shown in Figure 16.

**Figure 18:**
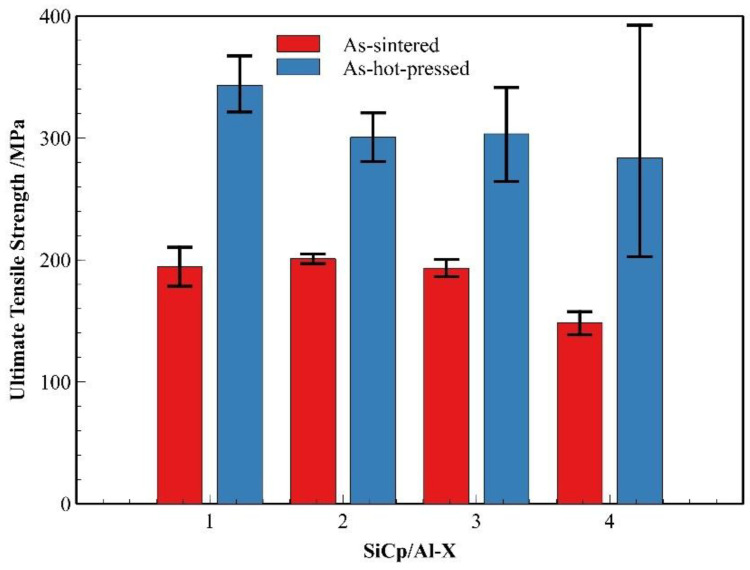
Comparison of strength between different SiC_p_/Al composites in the as-sintered and hot-pressed states.

Building upon the influence of reinforcement characteristics on composite densification, we further investigated the mechanical properties of the composites in both sintered and hot-pressed conditions. As shown in Figure 18, comparative analysis of SiC_p_/Al-1, SiC_p_/Al-3, and SiC_p_/Al-4 reveals that when the SiC content increases to 45%, the sintered composites exhibit a significant reduction in average strength. More notably, the hot-pressed composites demonstrate even more pronounced strength variations—while the average hot-pressed strength progressively decreases with rising ceramic content, the strength data simultaneously shows increasingly wider fluctuation ranges. The increased ceramic content substantially elevates the challenges for densification for composites in both processing states. Particularly in hot-pressed condition, where the achieved density directly governs material strength, a higher SiC content renders the composites more sensitive to the processing parameter variations. Fluctuation either in temperature or pressure during hot-pressing results in changes in the mechanical strength data, explaining the observed broadening of strength variations at elevated reinforcement levels. Furthermore, it can be observed that at the same ceramic content, the aluminum matrix composites prepared with finer SiC particles achieve higher tensile strength in the dense state.

**Figure 19:**
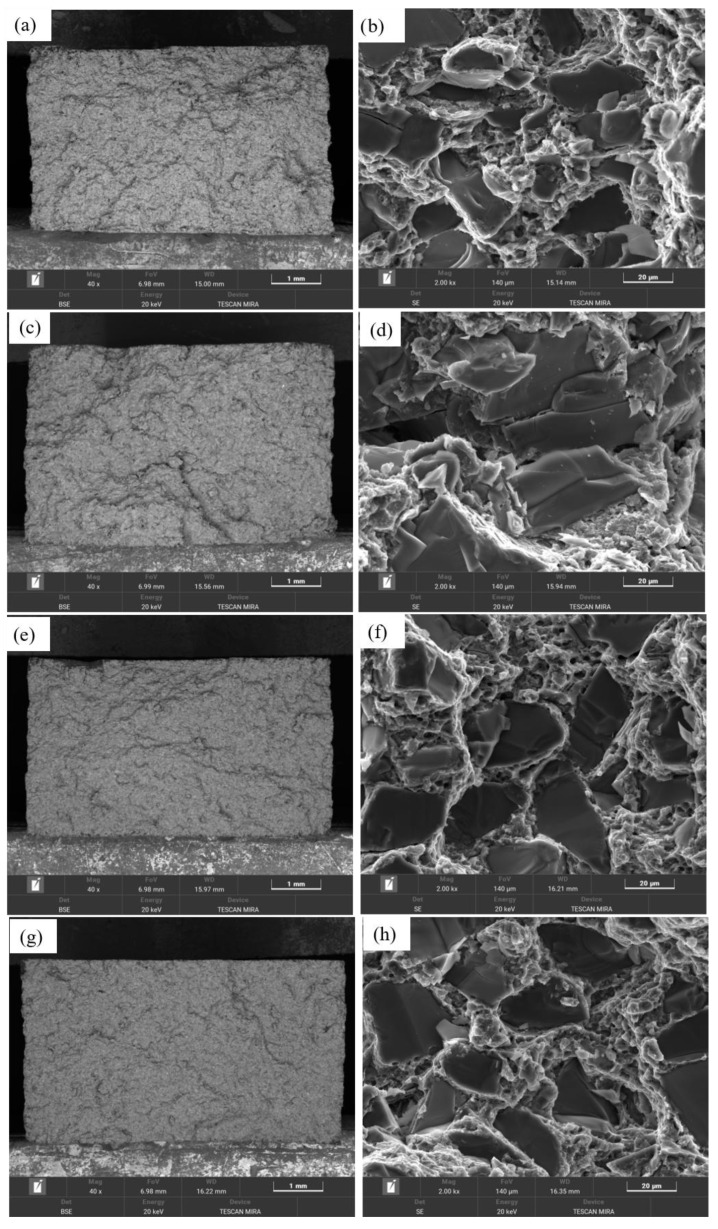
SEM images of fracture surfaces for different SiCp/Al composites in as-sintered and hot-pressed states: (**a**,**b**) SiCp/Al-1; (**c**,**d**) SiCp/Al-2; (**e**,**f**) SiCp/Al-3; (**g**,**h**) SiCp/Al-4.

A comparative analysis was conducted on morphologies of the fracture surfaces of different composite materials. Figure 19a,c,e,g shows low-magnification fracture images of the four composites, revealing distinct fracture characteristics at this scale. Comparing SiC_p_/Al-1, SiC_p_/Al-3, and SiC_p_/Al-4, the fracture surfaces became progressively smoother with increasing SiC content. In contrast, when comparing SiC_p_/Al-1 and SiC_p_/Al-2, the latter, which contains coarser SiC particles, exhibited more localized fragmentation. Figure 19b,d,f,h presents high-magnification SEM images of the fracture surfaces in the four composites, where fractured SiC particles were observed in all cases, with no significant evidence of particle pull-out. This indicates strong interfacial bonding between the SiC particles and the aluminum alloy matrix, allowing the SiC particles to effectively bear the load until their strength limit was exceeded, leading to particle fracture. Additionally, interfacial debonding was observed at the particle-matrix interfaces, while the matrix regions exhibited ductile dimples formed by plastic deformation and tearing. These findings suggest that the fracture failure modes of all four composites involved a combination of matrix ductile fracture and SiC particle fracture. Notably, the fracture surface of SiC_p_/Al-2, which contains coarse SiC particles, displayed prominent cracks that propagated through the SiC particles and extended into the matrix. Previous studies suggest that large SiC particles are more prone to defects, resulting in poor reinforcement efficiency, inferior mechanical properties, and weaker crack resistance compared to finer SiC particles [32,33].

## 4. Conclusions

Aluminum matrix composites were successfully fabricated using 2024 aluminum alloy powder and pure aluminum powder as matrix materials through conventional powder metallurgy combined with hot-pressing. The effects of various factors on the densification process were systematically investigated, yielding the following principal findings:

i. The sintered relative density of the composite first increased and then decreased with rising temperature and extended holding time, with the optimal sintering parameters identified as 600 °C for 30 min.
ii. As the densification process progresses, the microstructure of the composite undergoes significant changes, including the decomposition of divorced eutectic at particle boundaries into α-Al, θ phase (Al_2_Cu), and S phase (Al_2_CuMg); the formation of liquid phase by precipitates at particle boundaries promoting densification; and the precipitation of Mn/Fe-rich precipitates. Exceeding the optimal sintering parameter range leads to coarsening of precipitates and pores.
iii. In both the as-sintered and hot-pressed materials, Mg- and O-rich interparticle boundaries were observed. However, in the hot-pressed state, the concentration gradients of these elements decreased compared to the surrounding regions, indicating that hot pressing promotes a more homogeneous distribution of Mg and O.
iv. At the same SiC content, the aluminum matrix composites prepared with 31.9 μm SiC particles outperform those fabricated using 66.8 μm SiC. As the SiC content increases, the composites reinforced with 31.9 μm SiC exhibit a gradual decline in average tensile strength along with greater fluctuation in mechanical properties.

## Figures and Tables

**Table 1:** EDS results of a 2024 particle at the corresponding points in Figure 1b (wt.%).

Points	Al	Cu	Mg	Mn
1	78.40	15.81	4.44	1.35
2	91.90	4.00	2.57	1.53

**Table 2:** Composition of the Aluminum Matrix Composites.

	SiC/wt.%	D50 of SiC/μm	Theoretical Density/g/cm^3^
SiC_p_/Al-1	35	31.9	2.915
SiC_p_/Al-2	35	66.8	2.915
SiC_p_/Al-3	40	31.9	2.938
SiC_p_/Al-4	45	31.9	2.960

**Table 3:** EDS results of SiC_p_/Al-1 at the points corresponding to the locations in Figure 6a (wt.%).

Points	Al	Cu	Mg	Mn	Fe	Si	O
1	65.42	18.04	6.40	0.22	0.05	8.44	1.44
2	76.32	21.44	0.79	0.27	0.13	0.27	0.78
3	96.34	1.78	0.68	/	0.20	0.38	0.63

**Table 4:** EDS results of SiC_p_/Al-1 at the points corresponding to the locations in Figure 7b (wt.%).

Points	Al	Cu	Mg	Mn	Fe	Si	O
1	80.38	17.52	0.76	0.03	0.04	0.44	1.44
2	72.92	3.40	0.55	8.48	4.32	9.13	1.21
3	69.32	13.11	6.75	0.12	/	5.77	4.94

**Table 5:** EDS results of SiC_p_/Al-1 sintered at 600 °C with varying holding times (wt.%).

Point	Al	Cu	Mg	Mn	Fe	Si	O
Point 1	75.37	18.44	2.10	/	/	0.72	3.37
Point 2	63.41	29.45	1.62	/	/	0.83	4.69
Area 1	93.71	3.32	1.61	/	/	0.28	1.08
Point 3	55.26	36.32	1.05	/	/	0.24	7.13
Point 4	76.38	3.85	1.36	7.90	7.41	0.43	2.68
Point 5	93.11	1.29	2.03	0.36	0.14	0.29	2.78
Area 2	91.95	3.42	1.49	0.57	0.19	0.22	2.15
Point 6	61.81	4.23	0.52	13.49	10.36	7.66	1.93
Point 7	53.99	43.35	0.54	/	/	0.34	1.78
Point 8	94.47	2.71	1.30	0.55	0.24	0.34	0.38
Point 9	94.71	2.45	1.39	0.49	0.25	0.18	0.53
Point 10	63.79	4.03	0.51	12.36	10.41	7.18	1.72
Point 11	57.78	40.00	0.66	/	/	0.33	1.24

**Table 6:** EDS test results of SiC_p_/Al-1 hot-pressed samples at different temperatures (wt.%).

Points	Al	Cu	Mg	Mn	Fe	Si	O
1	67.28	3.84	0.65	11.12	9.66	6.32	1.12
2	73.68	23.45	1.07	0.10	0.01	0.35	1.35
3	69.41	1.45	15.67	0.22	/	9.87	3.38
4	74.91	1.00	9.72	0.29	0.06	9.49	4.54
5	61.98	35.02	1.25	0.04	/	0.63	1.08
6	62.55	5.66	0.47	13.95	9.97	6.68	0.72
7	74.61	22.91	0.79	0.25	/	0.44	1.00
8	65.98	3.16	0.41	11.33	8.45	9.76	0.92
9	53.66	1.59	6.27	0.79	0.25	28.28	9.41
10	73.20	3.80	1.03	11.23	3.83	5.75	1.17
11	78.96	18.20	1.51	/	0.01	0.24	1.08

## Data Availability

The original contributions presented in this study are included in the article/Supplementary Materials. Further inquiries can be directed to the corresponding author.

## Supplementary Materials

The following supporting information can be downloaded at: https://www.mdpi.com/article/10.3390/ma18215060/s1, Figure S1: Backscattered Electron Images (BEI) of SiC Particles: (a) SiC with D50 of 31.9 μm; (b) SiC with D50 of 66.8 μm; Figure S2: (a) Powder compaction die; (b) as-pressed sample; Figure S3: DSC curve of the green compact pressed from the matrix powder (a mixture of 2024 aluminum alloy powder and pure aluminum powder) material; Figure S4: Samples after hot pressing at different temperatures; Table S1: Chemical Composition of the 2024 Aluminum Alloy Powder (wt.%).
