# Supplementary material for: The Influence of Process Conditions and Reinforcement Characteristics on the Densification and Mechanical Properties of Powder Metallurgy SiCp/Al Composites
Source: Materials (Basel). 2025 Nov 6;18(21):5060. doi: 10.3390/ma18215060 (PMC12609148; doi:10.3390/ma18215060)
Supplement: Supplementary file 1 [file materials-18-05060-s001.zip › materials-3932787-supplementary.pdf]

Supporting information

The Influence of Process Conditions and Reinforcement Characteristics on the Densification and Mechanical Properties of Powder Metallurgy SiC_p/Al Composites

Liu-xu CAO[1], Qing-song DAI[2], Qi-wen LIANG[3], Xiao-yong ZHANG[1*]

1. State Key Laboratory of Powder Metallurgy, Central South University, Changsha 410083, China

2. Light Alloy Research Institute, Central South University, Changsha 410083, China

3. School of Materials Science and Engineering, Kunming University Of Science And Technology, Kunming 650093, China

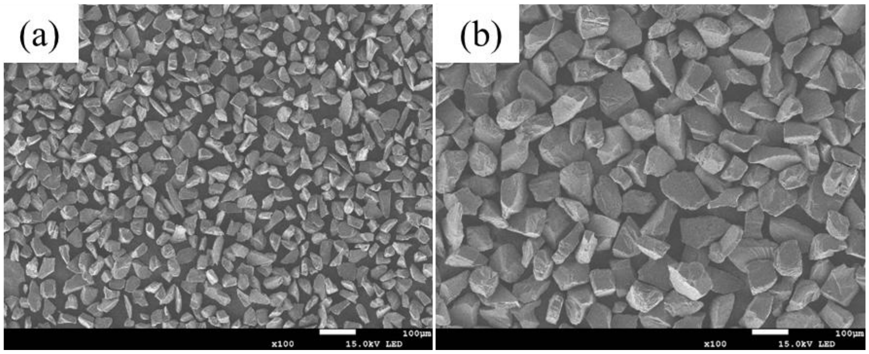

Figure S1. Backscattered Electron Images (BEI) of SiC Particles: (a) SiC with D50 of 31.9 μm; (b) SiC with D50 of 66.8 μm.

Table S1. Chemical Composition of the 2024 Aluminum Alloy Powder (wt.%).

Cu	Mg	Mn	Fe	Si	Zn	Cr	Ti	Al
4.28	1.55	0.47	0.11	0.084	＜0.05	＜0.01	＜0.001	Bal.

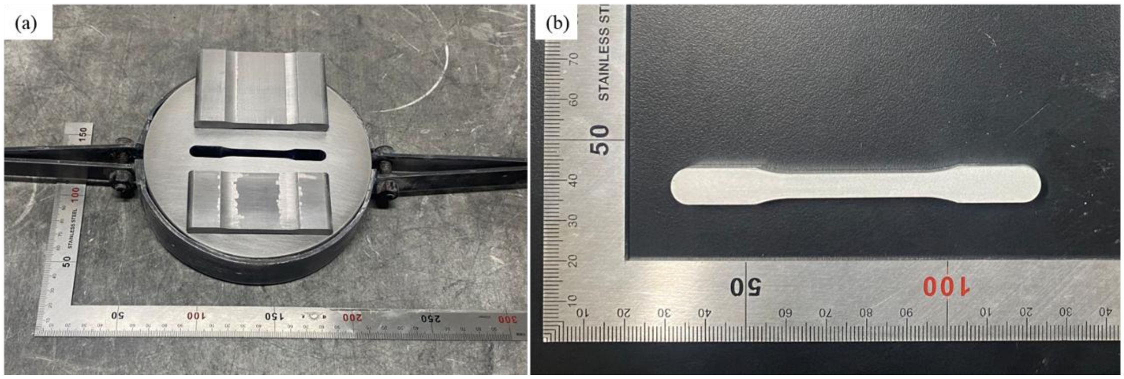

Figure S2. (a) Powder compaction die; (b) as-pressed sample.

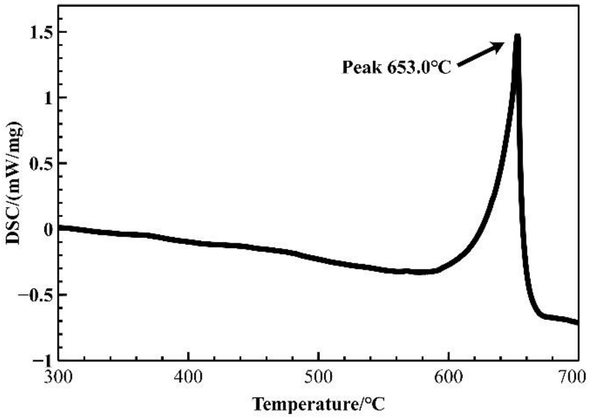

Figure S3. DSC curve of the green compact pressed from the matrix powder (a mixture of 2024 aluminum alloy powder and pure aluminum powder) material.

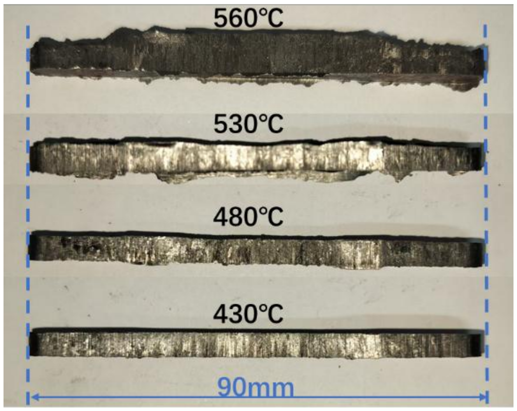

Figure S4. Samples after hot pressing at different temperatures.
